# Differential effect of alcohol on TNFα receptor II production in the presence of LPS challenge ex vivo

**DOI:** 10.1186/cc13422

**Published:** 2014-03-17

**Authors:** A Gavala, K Venetsanou, P Myrianthefs, E Manolis, C Kittas, G Baltopoulos

**Affiliations:** 1 ICU Agioi Anargyroi' General Hosrital, School of Nursing, Athens University, Athens, Greece; 2 Dentistry School, Athens University, Athens, Greece; 3 Medical School, Athens University, Athens, Greece

## Introduction

Acute alcohol exposure suppresses proinflammatory response, which may be related to increased susceptibility to infections [1]. The purpose of the study was to investigate the effect of acute exposure to alcohol on TNFα production capacity and TNFα receptors (TNFRs) in an ex vivo model of whole-blood stimulation with lipopolysaccharide (LPS).

## Methods

Whole blood was taken from healthy volunteers and was placed in tubes containing EDTA and immediately transferred to the laboratory. Heparinized blood samples were diluted 1:10 in RPMI 1640 culture medium (100 μl whole blood added in 900 μl RPMI 1640). Samples were preincubated with 0, 5, 12.5, 25, 50, 100 and 200 mM alcohol (EtOH) for 10 minutes at room temperature. After incubation, 500 pg LPS was added to each sample for 4 hours at 37°C. At the end of the process, samples were centrifuged (1,800 rpm, 5 minutes, r.t.). Culture supernatants were collected and stored at -70°C until measurements. TNFα and TNFR levels were determined in culture supernatant using the ELISA method [2].

## Results

**Figure 1:**
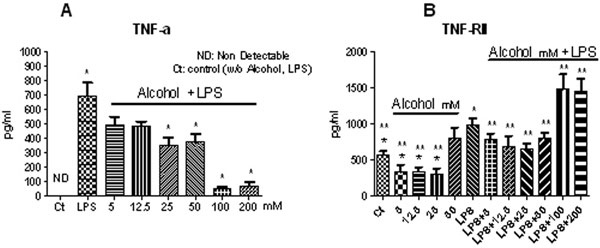


We studied 24 healthy males volunteers aged 36.5 ± 1.4 (X ± SEM). TNFα was not detected in samples treated without alcohol in the absence of LPS stimulation (control) or in the presence of alcohol alone (data not shown). TNFα production was significantly decreased at a dose of 25 mM alcohol after LPS stimulation (*P *< 0.0001) compared with LPS-challenged samples (Figure 1A). Alcohol had no effect on TNFR I production when incubated with or without LPS (data not shown). Alcohol at lower doses (<50 mM) seemed to decrease TNFR II levels, but an increase in TNFR II levels was observed at higher doses (>50 mM) of alcohol, which was statistically significant at doses of 100 and 200 mM alcohol after LPS stimulation ex vivo (*P *< 0.001) (Figure 1B).

## Conclusion

Our observations indicate a suppression of proinflam- matory response, but also a differential effect of alcohol on TNFR II production of whole blood in the presence of LPS challenge depending on the degree of alcohol intoxication.
